# Supplementary material for: Intermediate Hair Follicles from Patients with Female Pattern Hair Loss Are Associated with Nutrient Insufficiency and a Quiescent Metabolic Phenotype
Source: Nutrients. 2022 Aug 16;14(16):3357. doi: 10.3390/nu14163357 (PMC9416027; doi:10.3390/nu14163357)
Supplement: Supplementary file 1 [file nutrients-14-03357-s001.zip › nutrients-1853963-supplementary.pdf]

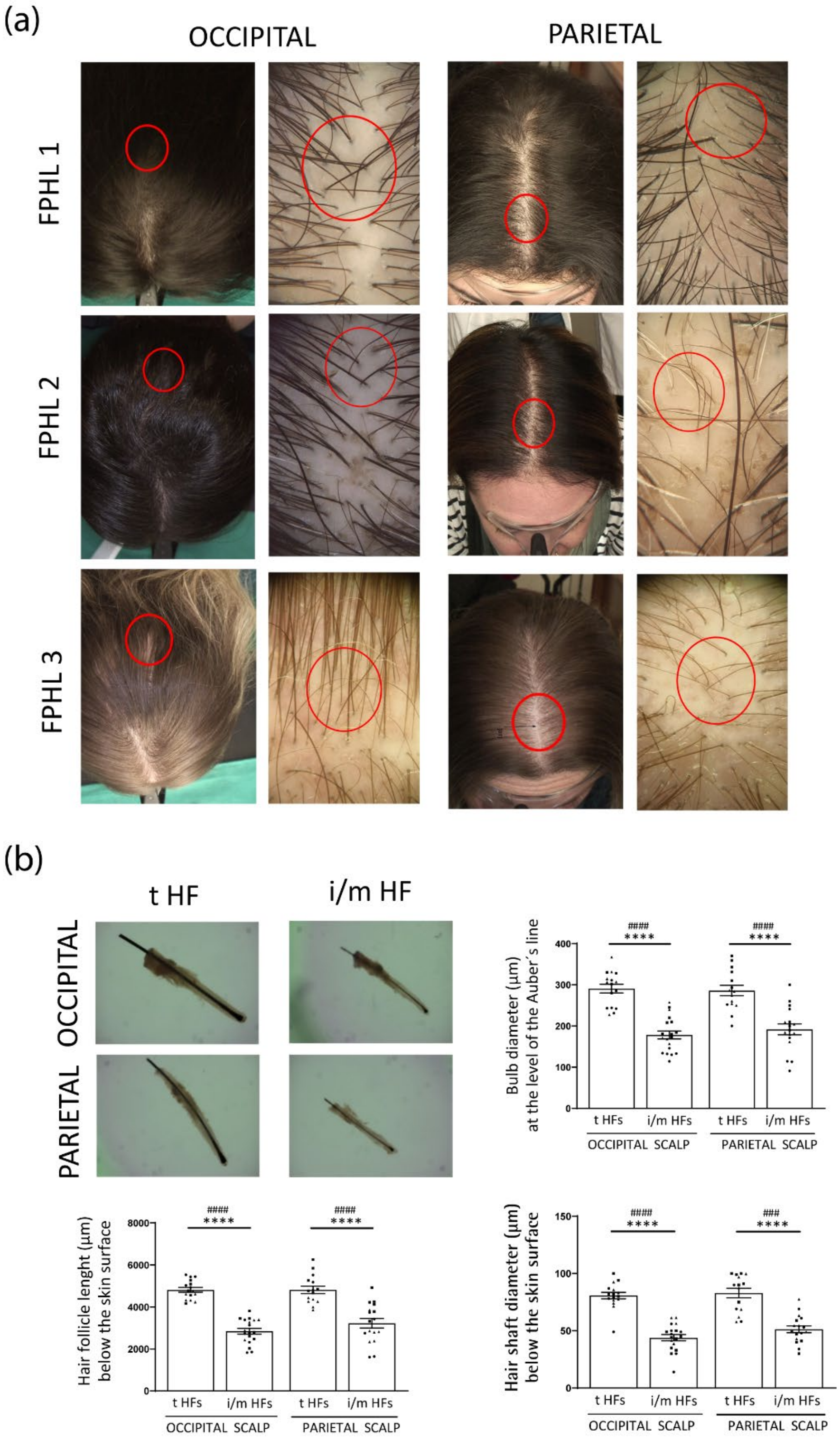

Figure S1. Clinical images and criteria to define intermediate FPHL HFs. (a) Representative clinical images of the parietal and occipital scalp from FPHL patients (patient 1, 2 and 3). Red circles highlight the regions from where skin punches for in situ analysis were obtained. (b) HF selection and pooling for metabolomic analysis and metabolic activity. After microdissection from FUEs, hair follicles were sorted,

pooled in four experimental groups (occipital terminal, occipital intermediate/miniaturized, parietal terminal, and parietal intermediate/miniaturized), based on the scalp origin and on bulb and hair shaft diameter, and hair follicle length measurements. As example, data from HFs used for metabolic analysis. Mean±SEM 15-19 HFs/experimental group from n=3 FPHL donors (squares, triangles, and circles), GraphPad 9.0. Kruskal-Wallis or Ordinary one-way ANOVA followed by Dunn's or Tukey's multiple comparison tests ###p<0.001, ####p<0.0001; Unpaired t-test or Mann-Whitney test **** p<0.0001

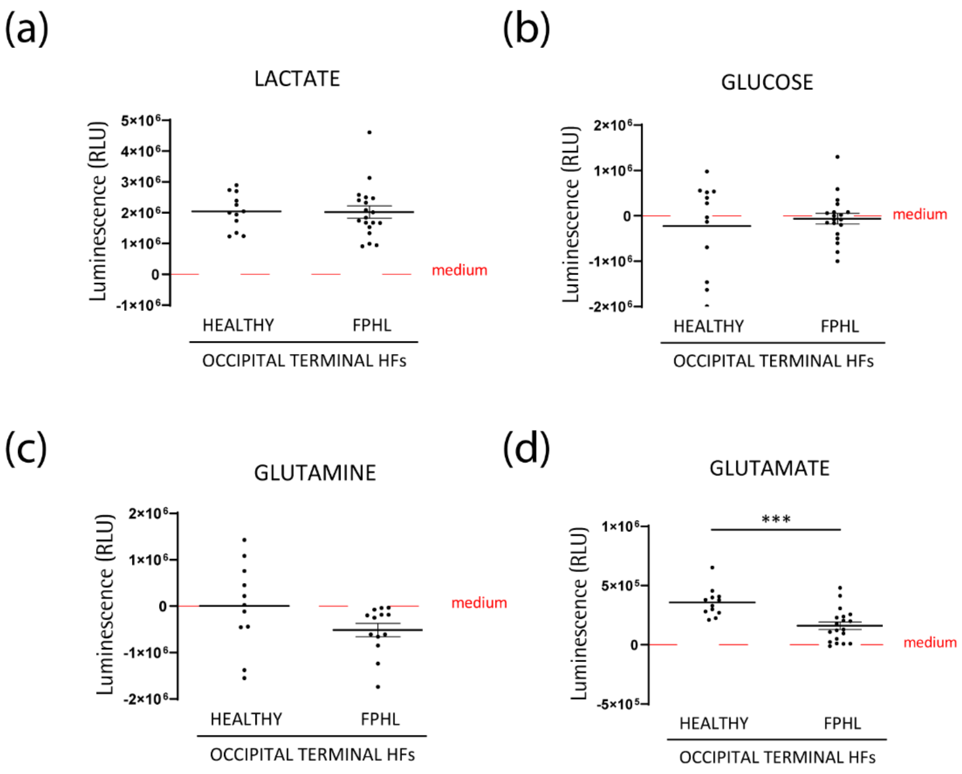

Figure S2. Comparison of metabolic activity of terminal occipital HFs from healthy donors and FPHL patients ex vivo. (a) Lactate, (b) Glucose, (c) Glutamine, and (d) Glutamate concentration was measured in the WCM culture medium after 24h HF culture following recommended protocols for Glo™ Assays kits (Promega®). Meam+SEM recorded luminescence from n=11-12 terminal occipital HFs/ group from n=3 healthy donors and n=13-19 terminal occipital HFs/ group n=3-4 FPHL donors. Red dotted lines indicate the amount in the blank, non-conditioned, WCM culture medium. GraphPad Prism 9.0; Mann-Whitney test, ****p<0.00001.

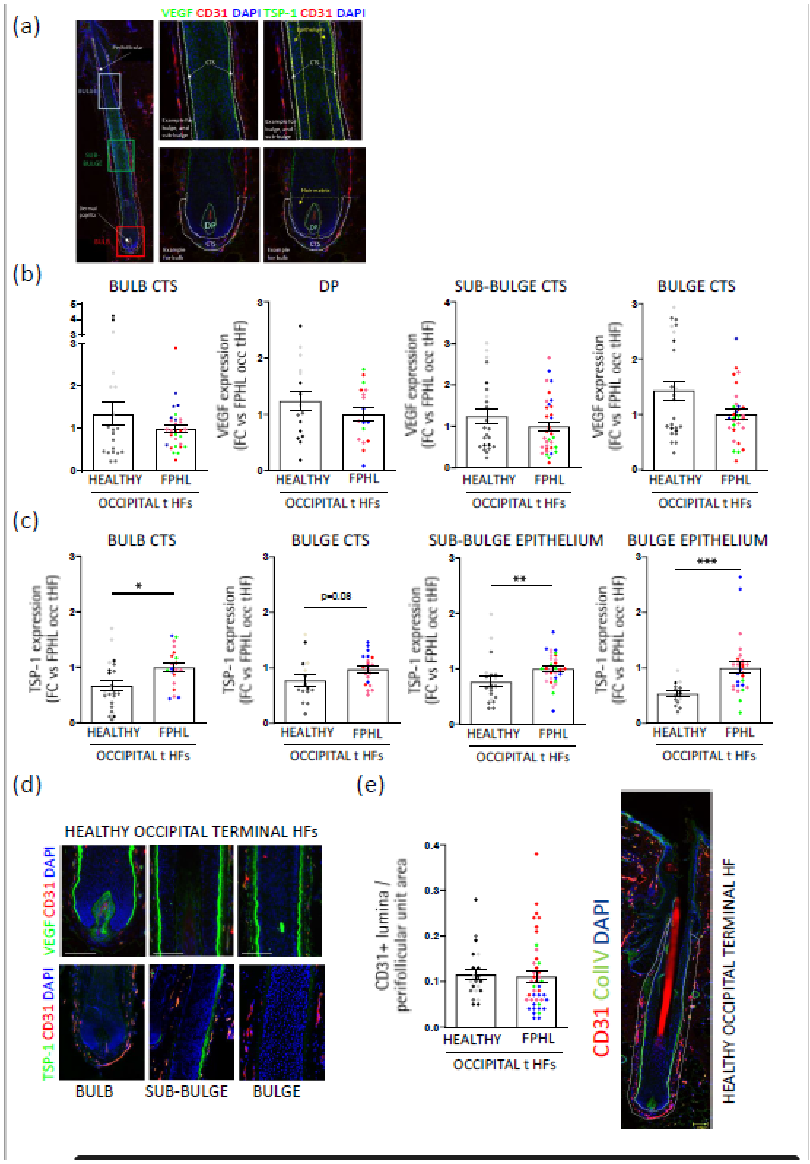

Figure S3. Terminal occipital HFs from FPHL patients express tendentially less VEGF and significantly more TSP-1 compared to terminal occipital HFs from healthy donors while the perifollicular vascularization is comparable.. HF pro- and anti-angiogenic factors expression and perifollicular vascularization in FPHL and healthy terminal occipital HFs. (a) Reference regions for quantitative analyses. (b) VEGF protein expression levels were measured by quantitative (immuno-)histomorphometry in the connective tissue sheath (CTS) of hair follicle bulb, sub-bulge and bulge regions and in the dermal papilla (DP) of occipital terminal (t) hair follicles from n=4 FPHL donors (1: red, 2: green, 3: blue, 5: pink) and n=3 healthy donors (1: black, 2: grey, 3: light grey). Mean±SEM from 16-34 hair follicles/group. GraphPad 9.0. Unpaired t-test or Mann Whitney test, n.s. (c) TSP-1 protein expression was measured by quantitative (immuno-)histomorphometry in the connective tissue sheath (CTS) of hair follicle bulb, and bulge and in the hair follicle epithelium at the sub-bulge and bulge level of occipital terminal (t) hair follicles from n=4 FPHL donors

(1: red, 2: green, 3: blue, 5: pink) and n=3 healthy donors (1: black, 2: grey, 3: light grey). Mean±SEM from 15-27 hair follicles/group. GraphPad 9.0. CTS: Unpaired t-test *p<0.05; epithelium: Mann Whitney test, *p<0.05, **p<0.01, ***p<0.001. (d) Representative images of in situ VEGF-CD31/CD31 and TSP-1/CD31 double immunofluorescence staining of bulb, sub-bulge, and bulge areas of a terminal hair follicle from FPHL and healthy occipital scalp skin. (e) The number of CD31+ lumina was counted in the perifollicular area of occipital terminal FPHL and healthy hair follicles. Mean±SEM n=22-43 HF from n=4 FPHL donors (1: red, 2: green, 3: blue, 5: pink) and n=3 healthy donors (1: black, 2: grey, 3: light grey). Graph Pad Prism 9. Mann-Whitney test, n.s. Representative images of in situ CD31/ColIV double immunofluorescence staining of an occipital terminal healthy hair follicle Dotted lines highlight the perifollicular areas.

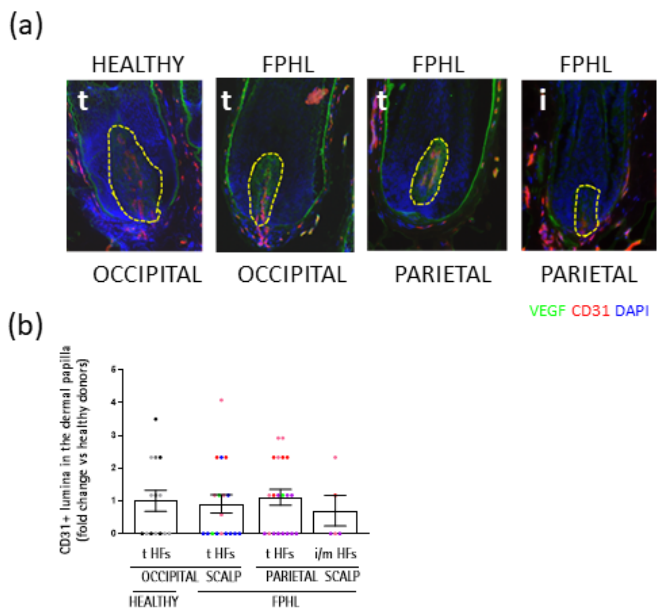

Figure S4. Dermal papilla vascularization is similar in healthy and FPHL hair follicles. (a) Representative images of in situ CD31/ColIV double immunofluorescence staining of the bulb region of occipital terminal hair follicles from healthy and FPHL occipital scalp skin, and of terminal and intermediate/miniaturized hair follicles from FPHL parietal scalp skin. Dotted lines highlight the dermal papilla. (b) The number of CD31+ lumina was counted in the hair follicles' dermal papilla. Mean±SEM n=5-20 HFs from n=3-5 FPHL donors (1: red, 2: green, 3: blue, 4: purple, 5: pink) and n=3 healthy donors (1: black, 2: grey, 3: light grey). Graph Pad Prism 9. Kruskal-Wallis test and Dunn's multiple comparison n.s.; Mann-Whitney test, n.s.
